# Schistosomiasis Involving the Central Nervous System: Case Report of a Rare Complication

**DOI:** 10.1155/2023/9968155

**Published:** 2023-12-12

**Authors:** Rahaf F. Alanazi, Maryam Al Karawi, Abdulrahman Almalki, Fahd Sufiani, Sarmad Al Karawi

**Affiliations:** ^1^College of Medicine, King Saud bin Abdulaziz University for Health Sciences, Riyadh, Saudi Arabia; ^2^King Abdullah International Medical Research Center, Riyadh, Saudi Arabia; ^3^College of Medicine, James Cook University, Townsville, Australia; ^4^Division of Neurosurgery, Department of Surgery, King Abdulaziz Medical City, Ministry of National Guard-Health Affairs, Riyadh, Saudi Arabia; ^5^Department of Pathology, King Abdulaziz Medical City, Ministry of National Guard-Health Affairs, Riyadh, Saudi Arabia

## Abstract

**Introduction:**

Schistosomiasis is a parasitic infection caused by schistosome invasion of blood circulation. Neuroschistosomiasis is a severe cerebral complication that accounts for less than 2.3% of reported cases. Patients present with progressive encephalitis, seizures, or both. Management includes antiparasitic medications, steroids, and surgical intervention. *Case Presentation*. We report a case of a 44-year-old female who presented to the ER with a history of transient loss of consciousness (LOC) and seizure. Radiological investigations revealed a right frontal brain lesion. Histopathological results confirmed the diagnosis of schistosomiasis.

**Conclusion:**

Schistosomiasis is a parasitic infection commonly diagnosed in patients who live in tropical areas. Early diagnosis with radiological and histopathological evaluation is required to identify patients at risk of developing severe neurological complications.

## 1. Introduction

Schistosomiasis, or bilharziasis, is a parasitic disease caused by haematogenic invasion with trematode worms of the genus Schistosoma [1, 2]. It is the second most common parasitic infection after malaria, mainly seen in tropical areas such as China, Japan, and the Philippines [1–3]. Around 700 million people are at risk of the infection, 207 million are affected worldwide, and 20 million develop severe clinical manifestations [1, 3, 4]. Central nervous system (CNS) involvement occurs in less than 5% [2]. We herein present a case of a 44-year-old Filipino female diagnosed with neuroschistosomiasis.

## 2. Case Presentation

A 44-year-old Filipino female was brought to the emergency department (ED) with a history of falls following focal myoclonus of the left lower limb for an unknown duration and transient LOC. She mentioned a left-sided headache and vomited twice in the ED. There is no history of fever, visual changes, loss of bowel or bladder control, fall, or trauma to the head.

On examination, her vital signs were within normal limits. She was alert and oriented, GCS 15/15, pupils were 3 mm and reactive, left orbital ecchymosis, intact vision and visual field, upward gaze palsy, left-sided facial weakness (UMNL), no swallowing difficulties, and no tongue deviation. Motor strength of the right upper limb (RUL) and right lower limb (RLL) was graded as 5/5, while the left upper limb (LUL) was graded as a 4/5 and left lower limb (LLL) graded as 4/5 proximally and 1/5 distally. Sensations of the limbs were intact to pain and light touch.

**Figure 1:**
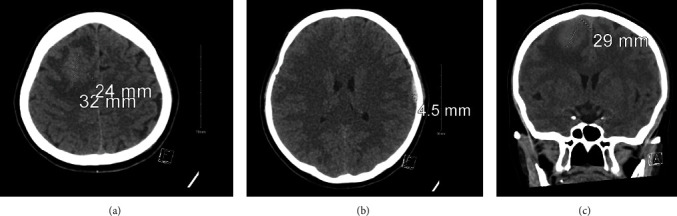
(a, b) CT brain axial view and (c) CT brain coronal view. (a, c) Right frontal subcortical hyperdensity with vasogenic edema and mass effect measuring 3.2 × 2.4 suggests a parenchymal hemorrhage. (b) Left temporal bone nondisplaced fracture with associated small epidural hematoma and periorbital fat stranding.

**Figure 2:**
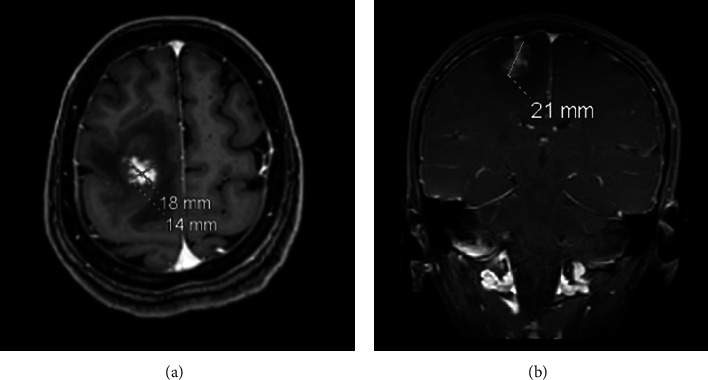
(a) Axial T1-weighted brain MRI with contrast. (b) Coronal T1-weighted brain MRI with contrast. (a, b) The solitary intra-axial nonhemorrhagic mass within the right frontal lobe measures 2.1 × 1.8 cm.

Radiological investigations were conducted to identify the underlying cause of her presentation. A computed tomography (CT) of the brain was taken on admission. It revealed a right frontal brain lesion with surrounding edema and a small left temporal extradural hemorrhage (EDH) with a nondisplaced fracture (Figure 1). A brain magnetic resonance imaging (MRI) with contrast was also done, and the findings revealed a solitary intra-axial mass within the right frontal lobe (Figure 2).

Considering the radiological findings, the patient underwent a right frontal craniotomy and debulking of the enhancing brain lesion under neuronavigation guidance. Multiple biopsies were sent for frozen section, permanent histopathology, and microscopic analysis of tissue cultures. The surgery was uneventful, and the patient tolerated the surgery well with no complications.

**Figure 3:**
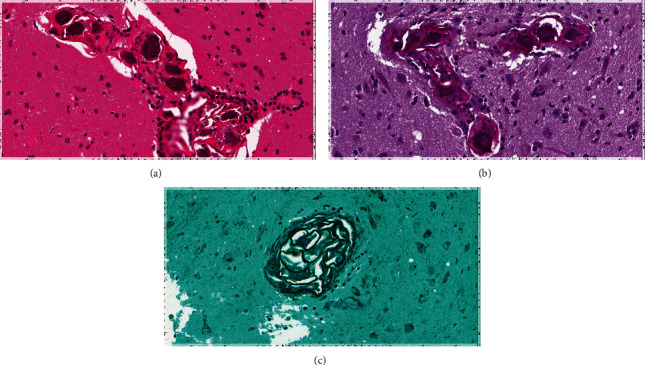
Cerebral schistosomiasis. Sections from brain cerebral cortex biopsies revealed scattered perivascular aggregates of parasitic eggs (outer shell and a terminal spine surrounding multiple nuclei). There is also gliosis with few perivascular lymphocytic cuffing. H&E, PASD, and GMS stains.

The histopathological findings reported scattered perivascular aggregates of parasitic eggs and gliosis, morphologically consistent with Schistosoma eggs (Figure 3).

**Figure 4:**
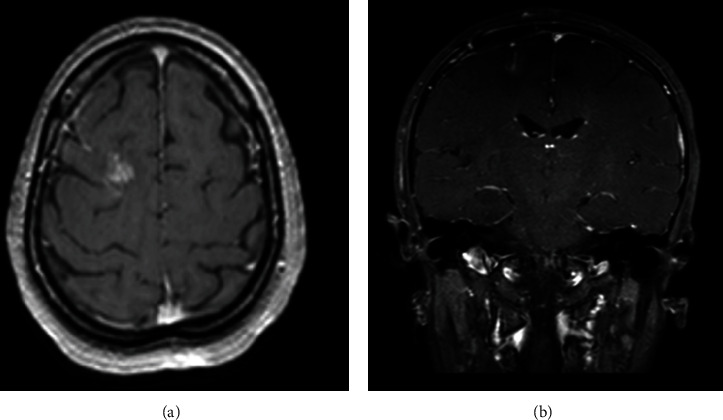
(a) Axial T1-weighted brain MRI with contrast. (b) Coronal T1-weighted brain MRI with contrast. (a, b) Improvement of the edematous change and mass effect associated with the right frontal lesion.

Considering the radiological and histopathological findings, the Infectious Disease team was consulted, and the patient was started on Praziquantel for 23 days, Albendazole for 19 days, and Prednisone for seven days. After completing the antiparasitic mediations and steroid course, she was discharged in stable condition with no neurological deficits; her gaze and left-sided weakness have dramatically improved. Repeated brain MRI after one month reported interval resolution of the enhancing lesion (Figure 4).

## 3. Discussion

Schistosomiasis is a parasitic infection that can reach the central nervous system (CNS) in severe cases. Their life cycle consists of two hosts: the intermediate host [2–4] (freshwater mollusks) and the definitive host (bloodstream of higher order vertebrate). Schistosome species have both asexual and sexual reproduction in snails and mammals. The asexual reproduction stage begins after infecting the intermediate host, a specific freshwater snail, to produce cercariae [1, 3, 4]. The cercariae are free swimming non-feeding, have forked tails, and use their endogenous nutrient stores for energy [2–4]. The infective cercariae use mechanical activity and produce proteolytic enzymes to penetrate human skin to complete their sexual reproduction. Afterward, they shed their tail and invade multiple organ systems by migrating through the venous circulation to mature in the liver eventually. After a short period, they reenter the blood circulation through retrograde venous flow in the Batson plexus, which connects the portal venous system and the vena cava to the spinal cord and cerebral veins [2–4]. They then migrate through the CSF, pair, and dispose of eggs in the CNS [2–5].

The pathogenesis of schistosomes is divided into two phases. Katayama syndrome is a hypersensitivity reaction that occurs within 14 to 84 days after the initial infection. The pathophysiology behind the clinical presentation was found to be due to the development of vasculitis and small vessel thrombosis by eosinophil-mediated toxicity [2–4]. The chronic phase is characterized by the maturation of the female and male worms, migration to the desired areas, and deposition of the eggs that begins after two months of the primary infection. When the eggs are disposed in the CNS, a cell-mediated immune reaction involves activating CD4+ T-helper cells, and granulomatous formation in the affected areas occurs. This explains the signs and symptoms of high intracranial pressure, radiculopathy, and subsequent neurological deficits due to the mass effect in the brain or spinal cord [2–4].

Neuroschistosomiasis is rare and was reported for approximately less than 5%; however, it causes significant morbidity and mortality in the developing world [3, 4]. Cerebral complications are more prevalent than the spine, accounting for 2.3% of all cases. The onset of the disease occurs within the primary infection, while 90% of the cases develop neurological deficits within two months. The most common features are seizures and encephalopathy. Other reported symptoms include fever, headache, nystagmus, speech disturbance, weakness, cranial nerve abnormalities, and papilledema. Hydrocephalus was reported when the cerebellum was involved [1–3]. A recent study investigating all published cerebral schistosomiasis cases' epidemiology, clinical presentation, and outcomes from 1989 to 2019 reported that 33 patients were included with a mean age of 28.2 years with male prevalence (*n* = 23, 69.7%). Most patients were from Brazil (*n* = 21, 63.6%); the commonly reported feature was seizure (*n* = 16, 48.5%). The diagnosis was made by histopathological investigations of brain tissue in 23 cases (69.7%), whereas the rest underwent serology and stool examination. Twenty-two cases showed complete resolution of symptoms (66.6%), two had hemianopsia, one had motor deficit, one had partial improvement, two were unreported, one lost follow-up, and one reported death [6].

Schistosomiasis invading the CNS was first described in 1889 [7]. It is believed that S. japonicum species commonly cause cerebral schistosomiasis as they are small and rounded, which makes them easily migrate to the brain, whereas S. mansoni species have been reported as the primary cause of spinal cord schistosomiasis. A case series study in Qatar reported three cases of Filipino men (ages 25 to 29) who presented with generalized tonic-clonic seizures. They underwent radiological and pathological investigations that showed case 1: brain MRI showed left anterior temporal lobe and frontal gyrus areas of high-signal intensity on T2/FLAIR, with a nodular and linear pattern of enhancement in contrast imaging, and histopathological examination showed brain parenchyma infiltrated by necrotizing granulomas harboring *Schistosoma* eggs with a positive serology for S. mansoni. Case 2 reported right parietal and left frontal and parieto-occipital subcortical mass lesions with hyperintensity on T2/FLAIR and hypointensity on T1 on brain MRI with underlying irregular spotty nodular dark signals in contrast images, with the histopathological investigations showing brain parenchyma with *Schistosoma* eggs surrounded by granulomatous inflammation. In case 3, they also reported three areas of abnormal signal intensity on T2/FLAIR involving the right cerebral hemisphere in the region of the frontal, occipital, and parieto-occipital lobes, associated with cortical thickening and postcontrast enhancement in brain MRI and brain parenchyma with *Schistosoma* eggs surrounded by exuberant granulomatous inflammation evident on histopathological examination [7]. However, in our case, we described a solitary intra-axial nonhemorrhagic mass within the right frontal lobe measuring 2.1 × 1.8 cm that was seen in a T1-weighted brain MRI with contrast and scattered perivascular aggregates of parasitic eggs (outer shell and a terminal spine surrounding multiple nuclei) and gliosis with few perivascular lymphocytic cuffing in sections from brain cerebral cortex biopsies. Although we found it essential to identify the specific type of Schistosoma egg found, it is challenging to distinguish between Schistosoma species based solely on the morphological features of their eggs, particularly after the formalin fixation and tissue processing, which were used in our case. These processes can obscure the subtle diagnostic features of the eggs. Nevertheless, given the patient's history and geographical location, it is most plausible that the infection was caused by Schistosoma japonicum. While the typical histological features of Schistosoma japonicum eggs, such as relatively small size, thin and colorless eggshell, and a characteristic lateral or terminal spine, were not definitively identified, we believe Schistosoma japonicum to be the most likely species involved in our case. All three cases from Qatar underwent cerebral biopsy alone, and once the diagnosis was confirmed, they were started on antifungal with favorable outcomes. However, our patient underwent surgical resection and then was started on an antifungal course. After finishing her management course, our patient was discharged with dramatic improvement.

Early recognition of schistosomiasis can provide patients with better outcomes and avoid severe complications. Neuroimaging and histopathological investigations should further assess neurological symptoms to achieve an accurate diagnosis [1, 2, 4]. In the acute phase, steroids with schistosomicidal drugs increase the cure rate and prevent the recurrence rate of symptoms, while in the chronic phase, surgery might be added for lesion resection [4].

## 4. Conclusion

Schistosomiasis is a parasitic infection that rarely presents with CNS involvement. Early diagnosis with radiological and histopathological evaluation is required to identify patients at risk of neuroschistosomiasis. Prompt surgical debridement targeted antischistosomal therapy, and steroids are the optimal combination of choice for managing such conditions to halt the invasive progression of the disease.
